# Genetic Landscape of Hearing Loss in Brazilian Patients Reveals Population‐Specific Variants and Clinical Correlations

**DOI:** 10.1111/cge.70186

**Published:** 2026-06-03

**Authors:** Stella Diogo‐Cavassana, Danillo Alencar‐Coutinho, Rafaella Abreu‐Oberhuber, Maria Eduarda Paramo‐Neto, Jeanne Oiticica, Ricardo Ferreira Bento, Ana Carla Batissoco, Karina Lezirovitz

**Affiliations:** ^1^ Laboratório de Otorrinolaringologia Genética Molecular, Celular e Translacional/LIM32, Hospital das Clínicas HCFMUSP, Faculdade de Medicina Universidade de São Paulo São Paulo Brazil; ^2^ Otorhinolaryngology Department Faculdade de Medicina da Universidade de São Paulo São Paulo Brazil

**Keywords:** Bartter syndrome, genetic hearing loss, Heimler syndrome, NGS, Usher syndrome, Waardenburg syndrome

## Abstract

Hearing loss (HL) is the most prevalent sensory disorder globally and a major public health challenge in Brazil, affecting over 1.5 million individuals. While over 150 HL‐associated genes have been identified, the genetic architecture in underrepresented populations remains poorly defined, often limiting diagnostic yield and precision medicine. We assessed the diagnostic performance of a comprehensive 218‐gene HL panel in 99 Brazilian probands (78 non‐syndromic; 21 syndromic) who had previously tested negative for *GJB2/GJB6* (DFNB1) and *MT‐RNR1* (m.1555A>G) and did not have ear malformations. Targeted next‐generation sequencing was followed by variant interpretation according to ACMG/AMP guidelines, segregation analysis, and longitudinal phenotypic re‐evaluation. Integration of Brazil‐specific allele‐frequency data was used to refine variant classification. A molecular diagnosis or a candidate variant was identified in 61 probands, yielding an overall diagnostic yield of 43%–62%, depending on classification stringency. We identified 19 novel variants across 15 genes, with *MYO7A* and *MYO15A* as the most frequently implicated. Notably, 10.4% of patients initially diagnosed with non‐syndromic HL carried pathogenic or likely pathogenic variants in syndromic genes (*PEX6, BSND, USH1C*, and *WFS1*), necessitating clinical reclassification. Segregation analysis and phenotypic reassessment further enabled the reclassification of three variants of uncertain significance (VUS). In syndromic cases, a molecular diagnosis was established in 41% of cases, including Usher, Waardenburg, Branchio‐oto‐renal, and Bartter syndromes. This first large‐scale clinical genetic evaluation of hearing loss in Brazil demonstrates that comprehensive gene panels incorporating population‐specific data significantly improve diagnostic accuracy. Our findings broaden the mutational landscape of HL‐associated genes, reinforce the value of integrated genetic approaches for underrepresented populations, and underscore their direct impact on patient care and clinical management.

## Graphical abstract

The Burden: Hearing loss (HL) is the most prevalent sensory disorder globally, affecting 1.5 million individuals in Brazil. The Gap: While > 150 genes are linked to HL, the genetic architecture in underrepresented populations like Brazil is poorly defined. The Problem: This lack of data limits diagnostic yield and the application of precision medicine. Study Group: 99 Brazilian Probands (78 non‐syndromic; 21 syndromic). Inclusion Criteria: Patients previously tested negative for *GJB2/GJB6* and *MT‐RNR1* (m.1555A>G) and had no ear malformations. Genetic Analysis: Targeted Next‐Generation Sequencing (NGS) using a comprehensive 218‐gene panel. Refinement Strategy: Variant interpretation via ACMG/AMP guidelines. Integration of Brazil‐specific allele‐frequency data. Segregation analysis and longitudinal phenotypic re‐evaluation. Overall Yield: Molecular diagnosis or candidate variant identified in 43%–62% of probands. Novelty: 19 novel variants discovered across 15 different genes. Frequent Drivers: *MYO7A* and *MYO15A* were identified as the most common genetic causes. Clinical Reclassification: 10.4% of “non‐syndromic” cases were reclassified after finding variants in syndromic genes (*PEX6, BSND, USH1C, WFS1*). 3 VUS (Variants of Uncertain Significance) were successfully reclassified through phenotypic reassessment. Syndromic Success: 41% diagnostic rate for cases including Usher, Waardenburg, BOR, and Bartter syndromes. Standard of Care: Comprehensive panels using population‐specific data significantly improve diagnostic accuracy. Scientific Contribution: This study broadens the known mutational landscape for hearing loss. Patient Outcome: Reinforces the value of integrated genetic approaches to directly improve clinical management and patient care in Brazil.

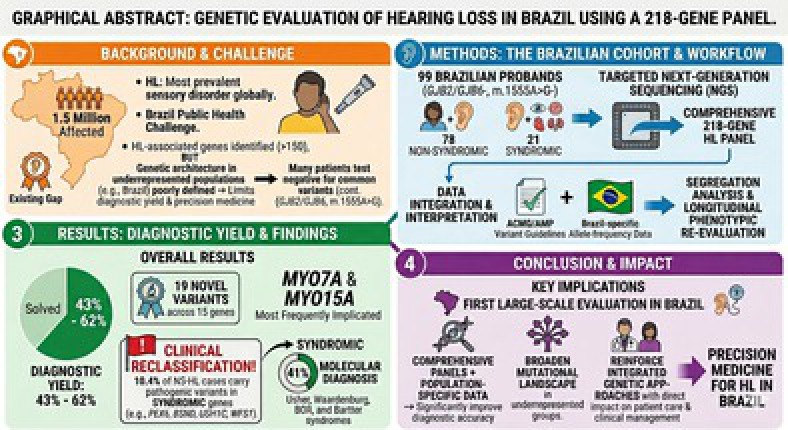

## 1 Introduction

Hearing loss (HL) is the most prevalent sensory deficit globally, affecting approximately 1.5 billion people, with over 430 million requiring rehabilitation services. In newborns, the incidence of permanent bilateral HL ranges from 1 to 4 per 1000 live births [1, 2, 3]. Approximately 50% to 60% of these cases have a genetic etiology, traditionally dichotomized into non‐syndromic (NS‐HL; ~70%) and syndromic (S‐HL; ~30%) forms. However, the molecular architecture of HL is remarkably complex; to date, more than 150 genes have been linked to NS‐HL, and over 400 syndromic conditions involve hearing impairment [4, 5].

The clinical utility of genetic testing has been revolutionized by Next‐Generation Sequencing (NGS), which enables simultaneous interrogation of hundreds of genes. While large‐scale studies in European and East Asian cohorts report diagnostic yields of ~40% [6, 7, 8, 9, 10, 11], these successes are not uniformly distributed worldwide. A significant “genomic divide” persists: individuals of African and Latin American descent are underrepresented in primary genomic databases by an estimated 20 to 30‐fold [12, 13, 14, 15]. This lack of diversity leads to a higher burden of Variants of Uncertain Significance (VUS) and limits the identification of population‐specific pathogenic alleles and founder effects [16, 17, 18].

In Brazil, the genetic landscape of HL is further complicated by extensive ethnic admixture involving Amerindian, European, and African ancestries [3]. While previous Brazilian studies have established the prevalence of common variants, such as those in the DFNB1 locus (*GJB2* and *GJB6*), the broader mutational spectrum remains poorly characterized in this heterogeneous population [15]. Furthermore, the boundaries between S‐HL and NS‐HL are increasingly blurred, as many “non‐syndromic” patients harbor variants in genes traditionally associated with syndromes, such as *USH2A* or *WFS1*, which may manifest with subtle or late‐onset extra‐auditory features [19, 20].

To address these gaps, we performed comprehensive genetic screening in an ethnically and clinically diverse cohort of 99 Brazilian probands using a targeted 218‐gene NGS panel. Our study sought to: (i) Quantify the diagnostic yield of a broad‐panel approach in an admixed population; (ii) Characterize the prevalence of “syndromic mimics” within seemingly non‐syndromic presentations; and (iii) Identify novel and recurrent variants to refine the mutational landscape of HL in Latin America.

By integrating rigorous ACMG/AMP (American College of Medical Genetics and Genomics/Association for Molecular Pathology) variant interpretation with deep phenotyping and segregation analysis, this work provides critical insights into the precision diagnosis and clinical management of hereditary hearing loss in underrepresented populations.

## 2 Subjects and Methods

### 2.1 Patient Recruitment and Ethical Considerations

Between 2013 and 2023, 619 individuals with HL were evaluated at the Otolaryngology/Genetic Deafness Clinic of the Hospital das Clínicas, University of São Paulo (HC–FMUSP). From this cohort, 100 unrelated probands were selected for targeted genomic analysis.

### 2.2 Inclusion/Exclusion Criteria

#### 2.2.1 Inclusion

Required a confirmed HL diagnosis and prior negative screening for pathogenic variants in *GJB2* , *GJB6* deletions (del(GJB6‐D13S1830) and del(GJB6‐D13S1854)), and the mitochondrial MT‐RNR1 m.1555A>G variant.

#### 2.2.2 Exclusion

Individuals with a previously established molecular diagnosis or structural inner ear malformations, as identified by computed tomography or magnetic resonance imaging, were excluded from the study. Cases with inner ear malformations had already been screened for *SLC26A4* variants in a prior study conducted by our group. Furthermore, *SLC26A4* ‐negative cases are currently being investigated in an ongoing study using customized whole‐exome sequencing to further clarify their genetic etiology. The study protocol was approved by the Ethics Committee of HC‐FMUSP and the Brazilian National Committee for Ethics in Research (CONEP). Written informed consent was obtained from all participants or their legal guardians. Pedigree analysis categorized inheritance as autosomal dominant (AD) (successive generations affected), autosomal recessive (AR) (affected siblings from unaffected parents or reported consanguinity), or sporadic (isolated cases with no relevant family history).

### 2.3 Clinical Phenotyping

Clinical data were extracted from electronic medical records. Audiological assessment included pure‐tone audiometry (air conduction: 250–8000 Hz; bone conduction: 500–4000 Hz) or, for pediatric/non‐cooperative patients, conditioned orientation response audiometry and/or auditory brainstem response (ABR) testing. Hearing severity was defined by the pure‐tone average (PTA) as follows: Mild: 20–40 dB HL, Moderate: 41–70 dB HL, Severe: 71–95 dB HL, Profound: > 95 dB HL. Age at onset was classified as prelingual (≤ 3 years) or postlingual (> 3 years).

### 2.4 DNA Preparation and Preliminary Genetic Screening

Genomic DNA was isolated from peripheral blood or saliva using a standard salting‐out protocol or commercial kits (Qiagen, Hilden, Germany). DNA quality and concentration were verified using a NanoDrop (Thermo Fisher Scientific, Waltham, MA, USA). Before NGS, all samples underwent baseline screening: Sanger sequencing for *GJB*2, multiplex PCR for *GJB6* deletions, and PCR–RFLP for the MT‐RNR1 m.1555A>G mutation.

### 2.5 Targeted Next‐Generation Sequencing (NGS)

Targeted enrichment was performed using a custom panel of 218 HL‐associated genes (BGI Genomics, Shenzhen, Guangdong, China), covering all coding exons and 20 bp of flanking intronic sequences. Bioinformatics Pipeline: Alignment: Raw FASTQ files were aligned to the human reference genome (GRCh37/hg19) using BWA‐MEM. Post‐processing: Duplicate reads were flagged with GATK MarkDuplicatesSpark, followed by base quality score recalibration (BQSR). Variant Calling: Single‐nucleotide variants (SNVs) and small insertions/deletions (indels) were called using GATK HaplotypeCaller. CNV Detection: Exon‐level copy number variants (CNVs) were identified using ExomeDepth. Visualization: All candidate variants were manually inspected using the Integrative Genomics Viewer (IGV).

### 2.6 Variant Annotation and Pathogenicity Interpretation

Variants were filtered for rarity using a minor allele frequency (MAF) threshold of < 1% in global (gnomAD, ExAC, 1000 Genomes) and regional (AbraOM Brazilian database) repositories. Pathogenicity assessment was conducted via the Franklin platform (QIAGEN, Venlo, Limburgo, Países Baixos (Holanda)), integrating in silico scores from REVEL, CADD (Phred), and SpliceAI. Final classification followed ACMG/AMP guidelines [21], refined by the ClinGen Hearing Loss Clinical Domain Working Group (HL CDWG) specifications [22]. Variants were considered causative if they showed: (i) Strong evidence of pathogenicity (*P* = pathogenic/LP = likely pathogenic), (ii) Consistency with the predicted mode of inheritance. (iii) Phenotypic alignment with established gene–disease associations.

### 2.7 Validation and Segregation Analysis

Candidate SNVs and indels were validated by Sanger sequencing. CNVs were confirmed via quantitative PCR (qPCR). When family members were available, segregation analysis was performed to strengthen variant classification. Primers were designed using Primer‐BLAST, and sequencing chromatograms were analyzed using BioEdit against Ensembl reference transcripts.

### 2.8 Statistical Analysis

Diagnostic yield (solved vs. unsolved) was compared across clinical and demographic subgroups using Fisher's exact test. Analyses were performed in GraphPad Prism v.10. Significance was set at *p* < 0.05.

## 3 Results

### 3.1 Cohort Characteristics and Sequencing Performance

**FIGURE 1:**
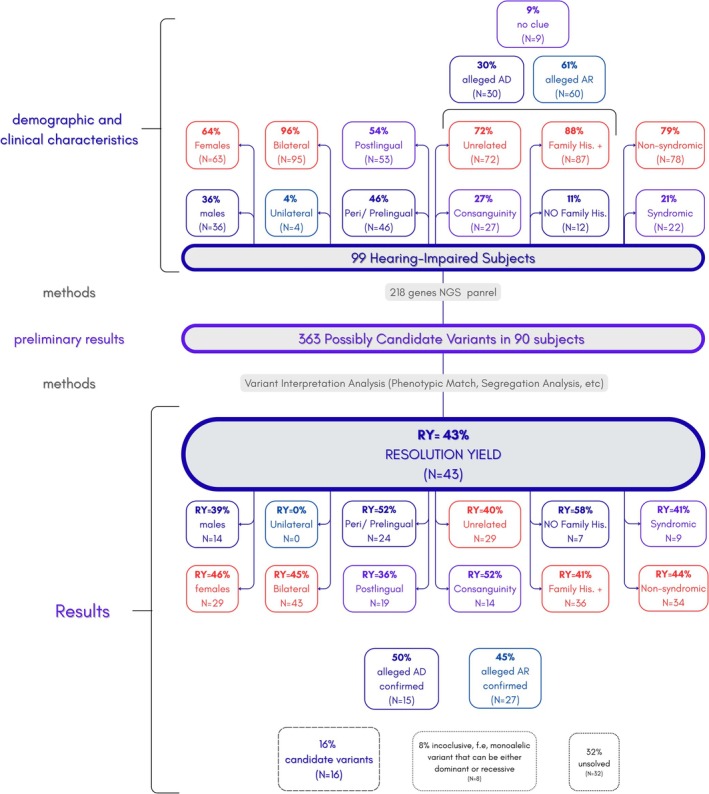
Overview of the clinical and demographic characteristics of the study cohort, including study design and diagnostic yield. [Colour figure can be viewed at wileyonlinelibrary.com]

The study initially recruited 100 unrelated probands. One sample failed quality control, leaving 99 individuals for the final analysis. The cohort was phenotypically diverse, though the majority presented with prelingual, bilateral sensorineural hearing loss (SN‐HL) and a positive family history (Figure 1, Table S1). All participants had previously screened negative for *GJB2/GJB6* variants and the mitochondrial MT‐RNR1 m.1555A>G mutation.

**FIGURE 2:**
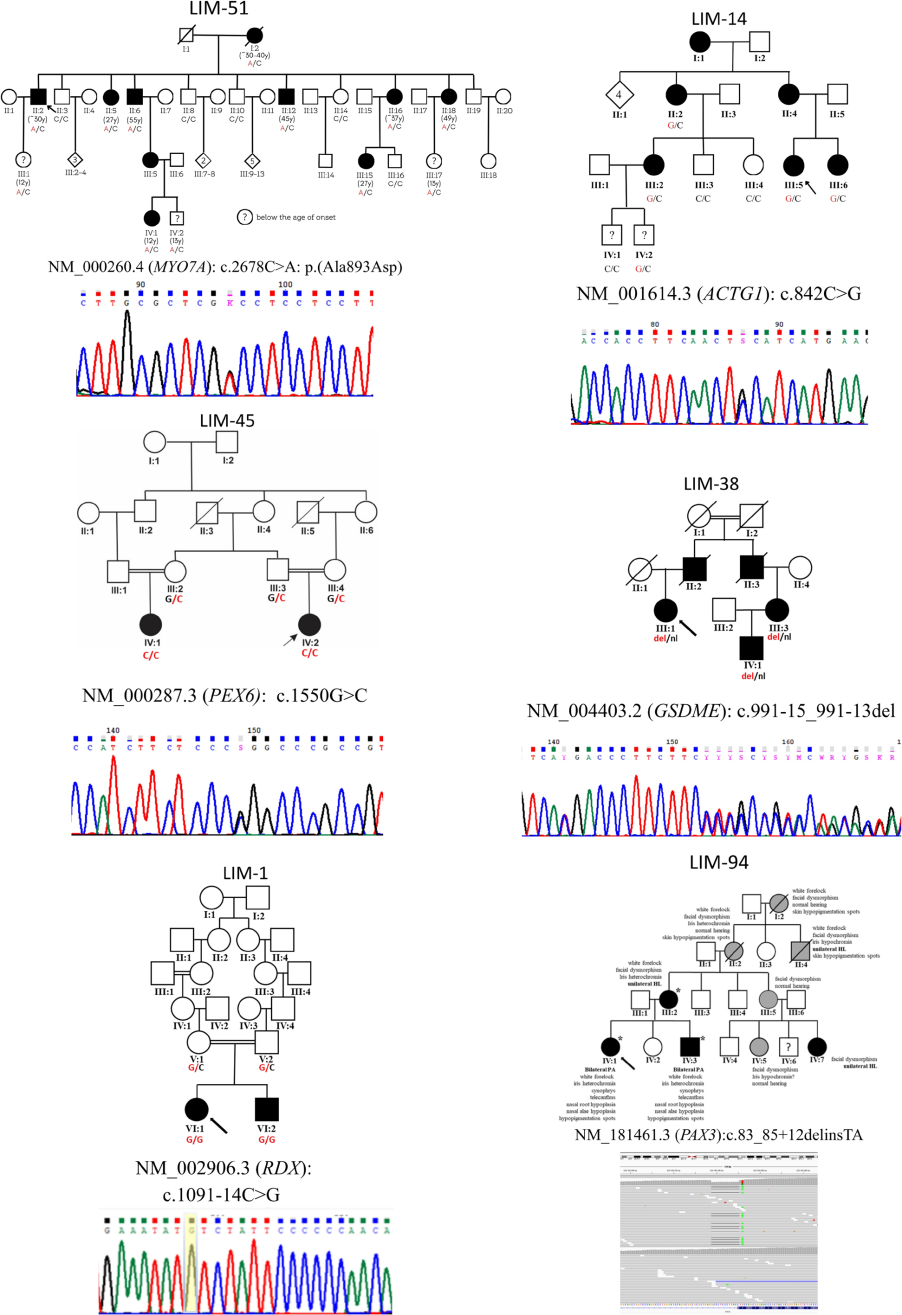
Pedigrees of resolved cases illustrating the impact of genomic findings on clinical diagnosis and variant classification. Case LIM‐51: The proband with postlingual progressive NSHL by age 30. Multiple affected maternal relatives shared a similar phenotype and carried a novel missense variant in *MYO7A*, which is likely pathogenic based on segregation data. Case LIM‐45: Representative case of phenotypic refinement. Initially diagnosed with NSHL, identification of a pathogenic variant in *PEX6* through NGS prompted re‐evaluation. Targeted follow‐up revealed enamel hypoplasia and nail abnormalities, leading to a revised diagnosis of Heimler syndrome. Case LIM‐14: Evidence supporting variant reclassification. A rare variant in *ACTG1* (MAF < 0.0001%), previously reported with conflicting interpretations in ClinVar, segregates with NSHL in four affected family members. Early‐onset presentation in individuals IV:1 and IV:2 provides strong evidence for classification as likely pathogenic. Case LIM‐1: Functional validation of a novel finding. A novel splice‐site variant in *RDX* was identified, with the pedigree supporting inheritance and functional assays confirming pathogenicity (manuscript in press). Case LIM‐38: Illustration of cohort‐specific mutational spectrum. This pedigree highlights a recurrent *GSDME* variant in the Brazilian population associated with postlingual progressive NSHL, underscoring its relevance for local diagnostic prioritization. Case 94: Clinical variability in syndromic hearing loss. A novel indel *PAX3* variant segregates in an affected family, demonstrating variable expressivity consistent with Waardenburg syndrome type I. [Colour figure can be viewed at wileyonlinelibrary.com]

Targeted sequencing of 218 HL–associated genes (7.16 Mb) achieved a mean target coverage of 99.99% and a mean depth of 300×. This depth provided high analytical sensitivity for SNVs, small indels, and exon‐level CNVs. Initial filtering identified 363 rare coding or splice‐site variants in 90.9% (90/99) of probands. Nineteen novel candidate variants were identified (Table 1, Figure 2).

**TABLE 1:** Summary details of the novel variants.

ClinVar, DVD, dbSNP	Genomic position (GRCh37)	Base change	Amino acid change	Exon on/intron	Zyg.	GnomAD (aggregated)/AbraOM	ClinVar, DVD, dbSNP	Aggregated score (Franklin)	PMID	Pathogen.	ACMG criteria	Case ID	Status
*MYO7A*, NM_000260.4	11:76909541‐76909666	EX34 Del	—	Exon 34	HET	N/A, N/A	N/A, N/A, N/A	—	—	**VUS**	[0.55] 1A, 2B, 2E, 3A, 4E	**31**	Resolved comp. Het w/c.6070C>T p.(Arg2024*)
	11:76891511	c.2678C>A	p.(Ala893Asp)	Exon 22	HET	N/A, N/A	N/A, N/A, N/A	0.84	—	LP	PM2, PP3_mod, PP1_str	**51**	Resolved
*MYO15A*, NM_016239.4	11:76900483	c.3598G>A	p.(Gly1200Ser)	Exon 28	HET	0.000001, N/A	N/A, N/A, rs1016139683	0.866	—	**VUS**	PM2, PP3_mod	**55**	Resolved
	17:18024561	c.2447G>A	p.(Arg816Gln)	Exon 2	HET	N/A, N/A	N/A, N/A	0.37	—	**VUS**	PM2	**69**	Candidate comp het w/c.4136T>C:p.(Leu1379Pro)
*KCNQ4*, NM_004700.4	1:41303984‐41327799	EX14 dup	—	Exon 14	HET	N/A, N/A	N/A, N/A, N/A	—	—	**VUS**	[0.3] 1A, 2K, 2L, 3A	**3**	candidate
*MYO6*, NM_004999.4	6:76623781‐76633423	EX34‐EX35E del	—	Exon 34–Exon 35	HET	N/A, N/A	N/A, N/A, N/A	—	—	**LP**	[0.9] 1A, 2D, 2B 3A	**72**	Resolved
	6:76570751‐76570752	c.1485_1486insGA	p.(Tyr496Aspfs*7)	Exon 15	HET	N/A, N/A	N/A, N/A, N/A	—	—	**LP**	PVS1, PM2	**82**	Resolved
*TMC1*, NM_138691.3	9:75445412	c.2175delA	p.(Ala726Argfs*9)	Exon 22	HOM	N/A, N/A	N/A, N/A, N/A	0.01	—	**LP**	PVS1, PM2	**16**	Resolved
*EYA1*, *NM_000503.6*	8:72127644‐72127661	c.1558_1575del	p.(Val520_Asn525del)	Exon 16	HET	N/A, N/A	N/A, N/A, N/A	—	—	**VUS**	PM2, PM4, PP4	**6**	Resolved
*MITF*, NM_000248.3	3:69986974‐69998319	EX2‐EX5 del	—	Exon 2–exon 5	HET	N/A, N/A	N/A, N/A, N/A	—	—	**P**	[1] 1A, 2E, 2B, 3A, 4E	**83**	Resolved
*PAX3, NM_181458.4*	2:223163238‐223163252	c.83_85+12delinsTA	—	Exon 1–intron 1	HET	N/A, N/A	N/A, N/A, N/A	—	—	**P**	PVS1, PM2, PP4	**94**	Resolved
*PTPRQ*, NM_001145026.2	12:81004376‐81004377	c.4891G>TA	p.(Glu1631*)	Exon 33	HET	N/A, N/A	N/A, N/A, N/A	—	—	**LP**	PVS1, PM2	**86**	Resolved(comp het w/c.1359+2T>C)
*RDX*, *NM_002906.4*	11:110108391	c.1091‐14C>G	—	Intron 10	HOM	N/A, N/A	N/A, N/A, N/A	0.78	—	**VUS**	PM2, PP3, PP1	**1**	Resolved
*USH2A*, NM_206933.4	1:216390723	c.3157+6T>C	—	Intron 15	HET	N/A, N/A	N/A, N/A, rs952449338	0.77	—	**VUS**	PM2, PP3, PM3_sup, PP4_sup	**17**	Resolved comp het w/c.1467delT:p.(His490Metfs*101)
*KITLG*, NM_000899.4	12:88510811‐88974055	EX1‐EX10 del	—	Exon 1–exon 10	HET	N/A, N/A	N/A, N/A, N/A	—	—	**VUS**	[0.68] 1A, 2B, 2C, 3A	**67**	Candidate (GUS/VUS)
LOXHD1, *NM_001384474.1*	18:44104707‐44104709	c.4702_4704del	p.(Lys1568del)	Exon 30	HET	N/A, N/A	N/A, N/A, rs1416298510	0.01	—	**VUS**	PM2, PM4	**44**	Candidate comp het w/c.3856G>A:p.(Gly1286Arg)
*MYH14*, NM_001145809.1	19:50805054	c.5606G>T	p.(Arg1869Leu)	Exon 40	HET	N/A, N/A	N/A, N/A, N/A	0.58	—	**VUS**	PM2	**37**	Candidate
	19:50774764	c.3133delC	p.(Leu1045Cysfs*11)	Exon 25	HET	N/A, N/A	N/A, N/A, N/A	0.01	—	**LP**	PVS1, PM2	**18**	Candidate
GPRASP2, NM_001004051.4	X:101971103‐101971104	c.1307_1330dup	p.(Ser443_Glu444insGlyGluGluAlaLy…)	Exon 5	HEMI	0, N/A	N/A, N/A, N/A	—	—	**VUS**	PM2	**91**	Candidate

*Note*: The “*” is part of the mutation nomenclature in this Table.

### 3.2 Diagnostic Yield and Variant Classification

Variants were classified according to ACMG/AMP guidelines, incorporating ClinGen Hearing Loss Clinical Domain Working Group (HL CDWG) specifications. Cases were stratified into four categories: Solved, Candidate, Inconclusive, and Unsolved (Figure 1; Table 2).

**TABLE 2:** Summary of clinical characteristics and identified genetic variants across resolved, candidate, and inconclusive cases.

Gene	Variant	Status	ClinVar ID	Comment	Case ID	Sex	Parental consang.	Onset	Lateral.	Severity	Syndromic	Familial	Alleged IP	Confirmed IP
*MYO7A*, NM_000260.4,NC_000011.9	c.6070C>T p.(Arg2024*)/EX34 Del (g.76909541_76909666del)	Resolved	43318	Novel	31	F	N	PRE	BI	Profound	Y	N	?	AR
	c.6026C>A:p.(Ala2009Asp) hetero	Inconclus.	802709	AR or AD SNV?	33	F	N	PRE	BI	Severe‐profound	N	Y	AR	?
	c.1798‐7_1800delinsATCGGCTGCT/c.4489G>C:p.(Gly1497Arg)	Resolved	929939/802708	—	50	M	N	PRE	BI	Profound	Y	Y	AR	AR
	c.2678C>A:p.(Ala893Asp) hetero	Resolved	—	Novel	51	M	N	POST	BI	Moderate	N	Y	AD	AD
	c.3598G>A:p.(Gly1200Ser) hetero	Resolved	—	Novel	55	M	Y	POST	BI	Profound (RE)/moderate‐severe (LE)	N	Y	AD/AR	AD
*MYO15A*, NM_016239.4	c.8183G>A: p.(Arg2728His)/c.762C>G: p.(Tyr254*)	Resolved	228276/2090747	—	20	F	N	POST	BI	Profound	N	Y	AR	AR
	c.5117G>T:p.(Gly1706Val)/c.6302T>C:p.(Leu2101Pro)	Resolved	—/1027555	—	49	F	N	PRE	BI	Profound	N	Y	AD/AR	AR
	c.6518C>T:p.(Ser2173Phe) homo	Resolved	—	—	64	M	Y	PRE	BI	Severe‐profound	N	N	AR	AR
	c.2447G>A:p.(Arg816Gln)/c.4136T>C:p.(Leu1379Pro)	Candidate	—	Novel/VUS	69	M	Y	PRE	BI	Severe‐profound	Y	Y	AR	AR?
*CDH23*, NM_022124.6, NC_000010.1	c.4021G>A:p.(Asp1341Asn)/c.3178C>T: p.(Arg1060Trp)	Candidate	4923/226437	VUS	10	F	N	PRE	BI	Profound	N	Y	AR	AR?
	c.3579+2T>C/c.8734G>A:p. (Gly2912Ser)	Resolved	1458365/2136889	—	32	F	N	PRE	BI	Moderate‐profound (RE)/severe‐profound (LE)	N	Y	AR	AR
	c.9319+1G>C/c.3579 + 2T>C	Resolved	1066040/1458365	—	52	M	N	PRE	BI	Severe‐profound	Y	N	?	AR
*KNCQ4*, NM_004700.4, NC_000001.10	g.4132779_41303984dup (exon 14 duplication hetero)	Candidate	—	Novel	3	F	Y	POST	BI	Profound	N	?	AD/AR	AD?
	c.806_808del:p.(Ser269del) hetero	Resolved	208366	—	7	F	N	POST	BI	Profound	N	Y	AR	AD
	c.701A>T: p.(His234Leu) hetero	Candidate*	1185583	*KNCQ4 or MYH9?*	53	M	N	POST	BI	Mild‐moderate	N	Y	?	AD?
*GSDME*, *NM_001127453.2*, NC_000007.13	c.991‐15_991‐13del hetero	Resolved	179997	—	25	F	N	POST	BI	Profound	N	Y	AD	AD
	c.991‐15_991‐13del hetero	Resolved	179997	—	38	F	N	POST	BI	Profound	N	Y	AD	AD
	c.1231C>T: p.(Gln411*) hetero	Candidate	—	VUS	89	F	Y	POST	BI	Profound	N	Y	AR	AD?
*CLDN14*, NM_001146079.2	c.291C>A:p.(Cys97*) homo	Resolved	3338644	—	34	M	Y	PRE	BI	Severe‐profound	N	Y	AR	AR
	c.488C>T: p.(Ala163Val) homo	Resolved	228519	—	2	F	Y	POST	BI	Profound	N	Y	AR	AR
*LRTOMT*, NM_001145308.5, NC_000011.9	c.358 + 4A>C homo	Resolved	179792	—	23	F	Y	PRE	BI	Profound	N	N	AR	AR
	c.242G>A:p.(Arg81Gln) homo	Resolved	543	—	29	M	Y	PRE	BI	Severe (RE)/profound (OE)	N	Y	AR	AR
*MYO6*, NM_004999.4, NC_000006.11	g.76623781_76633423del (EX34‐EX35E del hetero)	Resolved	—	Novel	72	F	N	POST	BI	N/A	N	Y	AD	AD
	c.1485_1486insGA:p.(Tyr496Aspfs*7) hetero	Resolved	—	Novel	82	F	N	POST	BI	Severe‐profound	N	Y	AD	AD
*OTOGL*, NM_001378609.3, NC_000012.11	c.6787C>T:p.(Arg2263*)/c.4543C>T:p.(Arg1515*)	Resolved	3256698/2628997	—	88	F	N	POST	BI	N/A	N	Y	AR	AR
	c.4543C>T:p.(Arg1515*)/c.6019+5G>A	Resolved	2628997/499791	—	92	F	N	PRE	BI	Moderate	N	Y	AR	AR
*P2RX2*, NM_170682.4	c.1057G>A: p.(Gly353Arg) hetero	Resolved	987153	—	40	M	N	POST	BI	Profound	N	Y	AD	AD
		Resolved	987153	—	41	M	N	POST	BI	Moderate‐severe	N	y	AD	AD
*TMC1*, NM_138691.3, NC_000009.11	c.2175delA:p.(Ala726Argfs*9) homo	Resolved	—	Novel	16	M	Y	PRE	BI	Profound	N	Y	AR	AR
	c.1762T>C:p.(Trp588Arg) homo	Resolved	1976353	—	24	M	Y	PRE	BI	Severe‐profound	N	N	AR	AR
	c.1763 + 3A>G hetero	Inconclus.#	47864	Undet. 2nd variant?	26	F	N	PRE	BI	Severe‐profound (RE)/profound (LE)	Y	Y	AR	?
*MYH9, NM_002473.6*	c.5338C>T:p.(Arg1780Trp) hetero	Candidate*	666863	*KNCQ4 or MYH9?*	53	M	N	POST	BI	Mild‐moderate	N	Y	AD	AD
	c.2507C>T:p.(Pro836Leu) hetero	Candidate	623109	?	63	F	N	POST	BI	Moderate‐profound	N	Y	AR	AD?
	c.2114G>A:p.(Arg705His) hetero	Resolved	14079	—	78	F	N	POST	BI	Severe‐profound	N	Y	AD	AD
*WFS1, NM_006005.3*	c.2051C>T:p.(Ala684Val) hetero	Resolved	30556	—	87	F	N	PRE	BI	Profound	N mimic	Y	AR	AD
	c.1941C>A:p.(Cys647Ter) hetero	Inconclus.	2300477	Undet. 2nd variant?	5	F	Y	POST	BI	Profound	N	Y	AR	?
	c.1831C>T:p.(Arg611Cys) hetero	Candidate*	907591	AR or AD SNV?	68	M	Y	POST	BI	Moderate	N mimic?	Y	AD/AR	AD?
*PEX6*, NM_000287.4	c.1550G>C:p.(Arg517Pro) homo	Resolved#	501464	—	45	F	Y	PRE	BI	Moderate‐profound	N mimic	Y	AR	AR
	c.1802G>A:p.(Arg601Gln)/c.2011G>A:p.(Gly671Ser)	Inconclus.	198709/1380923	Non‐syndromic up to 9yo	99	F	N	PRE	BI	Severe‐profound	N mimic?	Y	AR	AR?
*USH1C*, NM_153676.4, NC_000011.9	c.308G>A:p.(Arg103His) homo	Resolved	39427	DFNB18 or S. USHER (mimic)	21	F	N	PRE	BI	Profound	N mimic?	Y	AR	AR
	c.497‐3C>A homo	Candidate	505102	Below RP onset – DFNB18 or USH1C?	95	M	N	PRE	BI	Profound	N mimic?	Y	AR	AR?
*ACTG1*, NM_001614.5	c.842C>G: p.(Ser281Cys) hetero	Resolved	1065023	—	14	F	N	POST	BI	Profound	N	Y	AD	AD
*BSND*, NM_057176.3	c.139G>A:p.(Gly47Arg) homo	Resolved#	4387	—	43	F	N	PRE	BI	Profound	N mimic	Y	AR	AR
*CLRN1*, NM_001195794.1	c.189C>A:p.(Tyr63*) homo	Resolved	4397	—	47	F	Y	PRE	BI	Profound	Y	N	AR	AR
*EPS8*, NM_004447.6, NC_000012.11	c.986T>C:p.(Leu329Pro)/c.205‐8A>G	Resolved	2513233/560888	—	62	F	N	PRE	BI	Moderate	N	Y	AR	AR
*EYA4*, NM_004100.5	c.242G>A: p.(Trp81*) hetero	Resolved	1965243	—	97	F	N	POST	BI	N/A	N	Y	AD	AD
*EYA1*, NM_000503.6	c.1558_1575del p.(Val520_Asn525del) hetero	Resolved	—	Novel	6	M	N	POST	BI	Profound	Y	Y	AD	AD
*MITF*, NM_000248.3, NC_000003.11	g.69986974_69998319del (EX2‐EX5 del)	Resolved	—	Novel	83	F	N	PRE	BI	Profound	Y	Y	AD	AD
*MPZL2*, NM_005797.4	c.220C>T:p.(Gln74*) homo	Resolved	585269	—	42	F	Y	POST	BI	Profound	N	Y	AR	AR
*OTOF, NM_194248.3*	c.2153G>A: p.(Trp718*) homo	Resolved	48187	—	79	F	Y	PRE	BI	Moderate‐profound	N	Y	AR	AR
*PAX3*, NM_181458.4,NC_000002.11	c.83_85+12delinsTA hetero	Resolved	—	Novel	94	F	N	PRE	BI	Profound	Y	Y	AD	AD
*PCDH15*, *NM_033056.4*	c.733C>T:p.(Arg245*) homo	Resolved	4933	—	28	M	Y	PRE	BI	Profound	Y	Y	AR	AR
*PTPRQ*, NM_001145026.2, NC_000012.11	c.1359+2T>C/c.4891delinsTA: p.(Glu1631*)	Resolved	597495/—	Novel	86	F	N	POST	BI	Severe‐profound (RE)/moderate‐profound (LE)	N	Y	AR	AR
	c.2621C>A: p.(Ser874*) hetero	Inconclus.#	2582600	Undet. 2 variant?	27	M	N	POST	BI	Profound	N	Y	AR	?
*RDX*, NM_002906.4, NC_000011.9	c.1091‐14C>G homo	Resolved	—	Novel	1	F	N	PRE	BI	Severe‐profound	N	Y	AR	AR
*TMPRSS3 NM_001256317.3*	c.1180G>C: p.(.Asp394His) homo	Resolved	46099	—	19	F	Y	PRE	BI	Profound	N	N	AR	AR
*USH2A*, NM_206933.4, NC_000001.10	c.1467delT:p.(His490Metfs*101)/c.3157+6T>C	Resolved	1407399/—	Novel	17	M	N	POST	BI	N/A	Y	N	AD	AR
	c.2276G>T:p.(Cys759Phe) hetero	Inconclus.	2356	Undet. 2nd variant?	36	F	N	POST	BI	Severe	Y	Y	AR	?
*HARS2*, NM_012208.4	c.1439G>A:p.(Arg480His)/c.386G>A: p.(Arg129His)	Candidate	635270/3856775	GUS, VUS	90	F	N	POST	BI	Profound	N mimic?	Y	AR	AR?
*DNMT1, NM_001130823.3*	c.3458_3466dup: p.(Leu1155_Arg1156insProLysLeu) hetero	Candidate	801417	VUS	48	F	N	POST	BI	Profound	Y	Y	AR	AD?
*KARS1, NM_001130089.2*	c.1577C>T:p.(Ala526Val) homo	Candidate	694746	GUS, VUS	76	M	Y	PRE	BI	Severe‐profound	Y	N	AR	AR
*KITLG*, NM_000899.4, NC_000012.11	c.‐25482_*384629del (EX1‐EX10 del) hetero	Candidate	—	GUS, VUS, NOVEL	67	F	N	POST	BI	Mild (RE)/mild‐moderate (LE)	N	Y	AD	AD?
*LOXHD1, NM_001384474.1*	c.4702_4704del: p.(Lys1568del)/c.3856G>A:p.(Gly1286Arg)	Candidate	—/2199843	Novel, VUS	44	F	N	POST	BI	Moderate‐profound	N	Y	AR	AR?
*MYH14, NM_001145809.2*	c.3133delC: p.(Leu1045Cysfs*11) hetero	Candidate	—	Novel	18	F	N	PRE	BI	Profound	N	Y	AR	AD?
	c.5606G>T:p.(Arg1869Leu) hetero	Candidate	—	Novel	37	**F**	**N**	**POST**	**BI**	**Profound**	**N**	**Y**	**AD**	**AD?**
*TNC*, NM_002160.4	c.2006G>A:p.(Arg669His) hetero	Candidate*	3180116	GUS	68	M	Y	POST	BI	Moderate	N mimic	Y	AD/AR	AD?
*GPRASP2, NM_001004051.4*	c.1307_1330dup: p.(Ser443_Glu444insGlyGluGluAlaLy…) hemi	Candidate	—	Novel	91	M	N	PRE	BI	Severe‐profound	N	Y	AD	XL?
*TECTA*, NM_005422.4	c.2221A>T:p.(Lys741*) hetero	Inconclus.	—	Recessive variant?	71	M	N	PRE	BI	Moderate	N	Y	AR	?
*ADGRV1, NM_032119.4*	c.2612delG(p.Gly871Glufs*8) hetero	Inconclus.	1075638	Undet. 2^nd^ variant?	54	F	N	POST	BI	Mild‐moderate	N	Y	AD	?

*Note*: CASE ID 26, 27, 43 and 45: GJB2 monoallelic, 53: Two candidate variants (KNCQ4, MYH9), 68: Two candidate variants (TNC, WFS1).

Abbreviations: # Cases also monoallelic to *GJB2* recessive variants; * Cases with more than one candidate variant; AD, autosomal dominant; AR, autosomal recessive; Inconclus, inconclusive; POST, postlingual; PRE, prelingual.

Application of the interpretation pipeline identified 56 pathogenic (P) or likely pathogenic (LP) variants across 30 genes, and 31 variants of VUS across 25 genes. The outcomes were (Figure 1): Solved: 43.4% (43/99), Candidate: 16.2% (16/99), Inconclusive: 8.1% (899), Unsolved: 32.3% (32/99).

**FIGURE 3:**
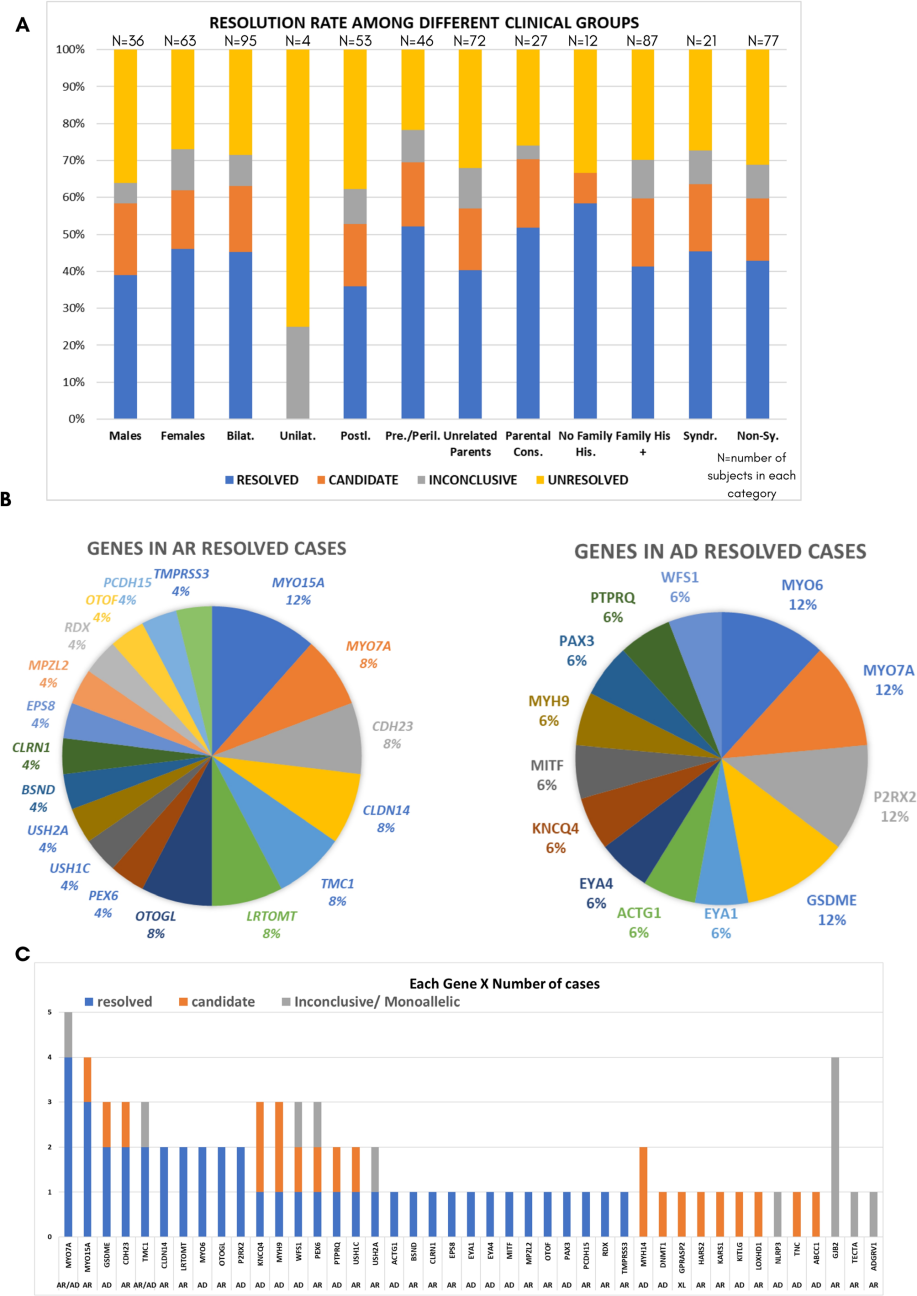
(A) Distribution of cases classified as resolved, candidate, inconclusive, and unresolved across different subject groups. (B) Number of cases attributed to each gene, stratified by inheritance pattern. (C) Number of cases per gene, categorized as resolved, candidate, or inconclusive. Some inconclusive cases harbor multiple variants potentially associated with the phenotype, while certain resolved or candidate cases also present monoallelic variants in additional genes; all such instances were classified as inconclusive/monoallelic. [Colour figure can be viewed at wileyonlinelibrary.com]

No statistically significant differences in diagnostic yield were observed across clinical or demographic subgroups, including age of onset, severity, or family history (Fisher's exact test, *p* > 0.05), demonstrating the panel's robust performance across heterogeneous presentations (Figure 3a).

### 3.3 Molecular Landscape of Resolved Cases

Of the 43 resolved cases, 17 (39.5%) involved genes associated with syndromic hearing loss, prompting clinical reassessment in those individuals (Table 2; Tables S1 and S2).

Regarding inheritance patterns, autosomal recessive (AR) predominated in 60.5% (26/43) of solved cases (Table 2; Tables S1 and S2). The most frequently implicated genes were *MYO15A*, *MYO7A*, *CDH23*, *CLDN14*, *LRTOMT*, *TMC1*, and *OTOGL* (Figure 3B,C). Six cases of Usher syndrome (*MYO7A*, *USH2A*, *CLRN1*, *PCDH15*, *CDH23*) and one case of Heimler syndrome type 2 (*PEX6*) were confirmed.

Autosomal dominant (AD) accounted for 39.5% (17/43) of cases (Table 2 and Table S2). Leading genes included *GSDME, MYO7A, MYO6*, and *P2RX2* (Figure 3B,C). Syndromic diagnoses included Waardenburg syndrome (*MITF, PAX3*) and Branchio‐otic syndrome type 1 (*EYA1*).

Regarding novelty and recurrence, 19 variants were novel and have not been evaluated in affected patients, are absent or very rare in the general population, and are listed in Table 1. They contributed to the elucidation of 12 cases (27.9%). Notably, four variants, *GSDME* c.991‐15_991‐13delTTC (Figure 2), *P2RX2* c.1057G>A (p.Gly353Arg), *OTOGL* c.4543C>T (p.Arg1515Ter), and *CDH23* c.3579 + 2C>T, were identified in multiple unrelated probands.

For *GSDME* c.991‐15_991‐13delTTC, the variant detected in patient 38 co‐occurred with the rs2269812 polymorphism, whereas the same variant in patient 25 did not. This finding supports a mutational hotspot rather than a founder effect, as previously suggested [23].

The *P2RX2* c.1057G>A variant was originally described in an Italian family, where it segregated with the phenotype, and functional studies supported its deleterious effect. In our cohort, however, both individuals harboring this variant are homozygous for rs4883632, and three of the four carriers reported in gnomAD share this background, suggesting a possible founder effect.

Additional analyses are required to clarify the basis for the recurrence of *OTOGL* c.4543C>T (p.Arg1515Ter) and *CDH23* c.3579 + 2C>T.

### 3.4 Candidate and Inconclusive Findings

In 16 cases (16.2%), candidate variants were identified but lacked sufficient evidence for definitive classification. In contrast to solved cases, AD inheritance was more frequent in this group (60%, 12/20), with *KCNQ4*, *MYH14*, and *MYH9* appearing repeatedly (Table 2, Table S1, Figure 3C).

Among the unresolved or partially explained cases, several variants deserve particular attention. A possible X‐linked inheritance pattern was observed in a male proband with postlingual unilateral SN‐HL who carried a likely causative variant in *COL4A6*. Pathogenicity was supported by segregation in his affected brother, although it remains classified as a VUS. Candidate variants were identified in *DNMT1* (OMIM* 126375; Ataxia, Deafness, and Narcolepsy syndrome) and *KARS1* (OMIM* 601421, developmental delay and hypotonia), but lacked segregation data or full phenotypic matching to confirm (Table 2, Table S1).

Eight cases were deemed inconclusive/unsolved due to monoallelic P/LP variants in genes where a second hit was missing (*GJB2, USH2A, ADGRV1*) or where the inheritance mode (AD vs. AR) could not be determined (*TMC1, PTPRQ, MYO7A, TECTA*) (Table 2, Table S1; Figure 3C). The remaining 30 cases were unsolved, including nine individuals with no rare variants detected in the 218 targeted genes.

## 4 Discussion

This study underscores the profound genetic heterogeneity of HL in the Brazilian population and demonstrates the high clinical utility of comprehensive gene panel testing. Our diagnostic yield ranged from 43.4% (under conservative criteria) to 63.6% (including candidate cases), aligning with or exceeding international benchmarks [6, 7, 10]. These findings support integrating deep phenotyping and segregation analysis into routine diagnostics to improve variant interpretation and diagnostic resolution.

### 4.1 Ancestry and the “Diagnostic Gap” in Admixed Populations

The Brazilian population is a genetic mosaic of Amerindian, European, and African ancestries [3]. This complexity directly affects variant interpretation, as many identified alleles were novel or absent from global databases such as gnomAD [16, 17, 18]. Our results contrast sharply with studies in less diverse or underrepresented cohorts Sloan‐Heggen et al. [6]; for instance, while Yan et al. [10] reported a 0% diagnostic rate in a Guatemalan cohort using a 180‐gene panel, our higher resolution rate likely reflects the inclusion of 218 genes and the use of regional reference data.

The exclusion of DFNB1‐positive cases in our recruitment, a group that accounts for ~13.1% of Brazilian HL [3, 15], allowed us to uncover a broader mutational spectrum. By “filtering out” the most common genetic cause, we enriched our cohort for rarer etiologies in genes such as *PEX6, MPZL2, EPS8*, and *BSND*, many of which were not covered in previous Brazilian studies [24]. Relative to the Brazilian cohort analyzed by Antunes et al. [24], which reported a 36.7% diagnostic rate (39% using a targeted panel and 22% by whole‐exome sequencing), the present study achieved a higher yield (44%).

By comparison, Baux et al. [25] achieved a 48% diagnostic rate in French subjects with non‐syndromic hearing loss using a panel‐based approach without pre‐screening, a figure likely inflated by the inclusion of DFNB1‐related cases. Indeed, the contribution of DFNB1 to hearing loss is well documented, accounting for 13.1% of Brazilian cases, 14% in Japanese, and 24% in the French cohort, reflecting its higher prevalence among populations with greater European ancestry [13].

### 4.2 The Blur Between Syndromic (S‐HL) and Non‐Syndromic (NS‐HL)

Among syndromic cases with confirmed molecular diagnoses, Usher syndrome (OMIM# 276900) was the most frequent (six probands), consistent with its status as the leading cause of deaf‐blindness and the second most common S‐HL [26, 27] after Pendred syndrome (OMIM# 274600). Four cases corresponded to Usher type I (*MYO7A, PCDH15, CDH23*), characterized by prelingual profound S‐HL and early‐onset retinitis pigmentosa, whereas one case was compatible with Usher type II (*USH2A*, OMIM # 276901) and presented postlingual progressive HL [26]. A proband with a homozygous nonsense variant in CLRN1 (OMIM# 276902) exhibited an atypically severe phenotype resembling Usher type I, illustrating genotype–phenotype variability [28]. These correlations have potential therapeutic relevance, as certain genotypes, such as *PCDH15*‐related to Usher Syndrome type I (OMIM# 601067), may influence cochlear implant outcomes, underscoring the importance of molecular diagnosis for clinical counseling and management [29, 30].

Other confirmed syndromic diagnoses included Branchio‐otic syndrome (*EYA1* #OMIMI* 601653) [31], Waardenburg syndrome type I (*PAX3* OMIM# 193500) and II (*MITF* OMIM# 193510) [32], and Heimler syndrome type II (OMIM# 616617 *PEX6*) [33], highlighting the value of genetic testing in refining clinical diagnosis and guiding care.

Notably, several individuals initially classified as having NS‐HL were found to carry variants in genes typically associated with syndromic conditions (*BSND, PEX6, USH1C, WFS1*), illustrating the phenomenon of “non‐syndromic mimics,” in which HL may precede systemic manifestations [6, 34]. For example, two probands carrying biallelic *USH1C* variants presented with isolated congenital deafness and no current ophthalmological findings, but remain at risk for developing retinitis pigmentosa and vestibular dysfunction. At the last follow‐up, patient 21 was 16 years old and patient 95 was 6 years old; therefore, both may be more appropriately classified as having non‐syndromic DFNB18 rather than Usher syndrome [35, 36].

In another case, a homozygous *BSND* variant was consistent with Bartter syndrome type 4A and was retrospectively supported by prenatal polyhydramnios [37]. Likewise, biallelic *PEX6* variants were compatible with Heimler syndrome, with dental anomalies becoming apparent during follow‐up, whereas compound heterozygous candidate *HARS2* variants were identified in a female with progressive HL, warranting surveillance for ovarian insufficiency consistent with Perrault syndrome. In addition, heterozygous *WFS1* variants associated with Wolfram‐like syndrome [38] were detected in individuals with hearing loss, supported in one case by a family history of diabetes and late‐onset hearing impairment. Notably, patient 87 (*WFS1*: c.2051C>T: p.(Ala684Val)), whose variant was absent in both normal‐hearing parents, presented with profound pan‐frequency hearing loss, deviating from the characteristic low‐frequency or upsloping audiometric configuration associated with this gene [39]. Patient 5 is likely a monoallelic case involving *WFS1*, given that the p.(Cys647Ter) variant has previously been described in Brazilian patients in compound heterozygosity in association with Wolfram syndrome [40]. Usher syndrome, the leading genetic cause of deaf blindness, was the most frequent syndromic diagnosis in our cohort. The identification of biallelic *USH1C* variants in individuals presenting with apparently isolated congenital HL illustrates how molecular diagnosis may precede the onset of ophthalmologic manifestations, providing a critical “window of opportunity” for anticipatory care and early ophthalmologic surveillance before the development of retinitis pigmentosa, while also contributing to the understanding of genotype–phenotype correlations in both syndromic and non‐syndromic DFNB18 [41, 42]. Similarly, the detection of *HARS2* variants associated with Perrault syndrome [43] and *PEX6* variants linked to Heimler syndrome in patients with apparently isolated HL further demonstrates the predictive value of genomic testing, allowing clinicians to anticipate potential late‐onset manifestations, such as ovarian insufficiency in Perrault syndrome or dental enamel abnormalities in Heimler syndrome, and enabling proactive multidisciplinary management and long‐term clinical monitoring [44].

Together, these findings demonstrate how molecular diagnosis can precede syndromic recognition, enabling anticipatory care, targeted surveillance, and personalized genetic counseling. Consistent with this observation, nearly half of the solved cases in our cohort involved genes traditionally classified as syndromic, emphasizing the clinical relevance of genomic testing in individuals initially presenting with apparently non‐syndromic hearing loss [19].

### 4.3 Molecular Landscape and Recurrent Variants

The recurrence of specific variants, such as *GSDME* c.991‐15_991‐13delTTC and *P2RX2* c.1057G>A, across unrelated probands suggests potential regional founder effects within Brazil population. Genes encoding myosin motor proteins (*MYO7A, MYO15A*) and ion channel proteins (*KCNQ4*) were among the most frequently implicated in our cohort, showing consistent genotype–phenotype correlations, particularly regarding age of onset and progression.

The repeated identification of variants in genes associated with autosomal dominant hearing loss, including *GSDME, P2RX2*, and *KCNQ4*, may reflect either shared ancestral alleles or mutational hotspots that contribute to recurrent pathogenic variants across different families. Given the highly admixed genetic background of the Brazilian population, identifying such recurrent variants may provide important insights into population‐specific mutational spectra and improve variant prioritization in diagnostic pipelines [3, 45].

The identification of 18 novel variants significantly expands the known mutational landscape of genetic HL in Latin American populations. Following the ACMG/AMP guidelines and ClinGen HL CDWG specifications, we were able to upgrade several variants from VUS to Likely Pathogenic through segregation analysis. These findings reinforce the necessity of testing additional family members to resolve inconclusive NGS leads and improve diagnostic interpretation.

Importantly, the identification of recurrent and novel variants in this cohort highlights the need to expand population‐specific variant databases for underrepresented populations, which remains a major challenge for variant interpretation in clinical genomics.

### 4.4 Limitations and Future Directions

Despite the high yield, 30.3% of cases remained unsolved. These “genomic cold cases” likely stem from:

1. **Structural Variants:** Standard NGS panels struggle with CNVs in complex regions like *STRC* and *OTOA*.
2. **Non‐coding Mutations:** Deep intronic variants or regulatory element mutations are not captured by targeted exonic sequencing.
3. **Novel Genes:** Some patients may harbor mutations in genes not yet associated with HL.

For these individuals, trio‐based Whole Genome Sequencing (WGS) or transcriptomic analysis represents the next logical step in the diagnostic odyssey.

## 5 Conclusion

This study represents the largest and most comprehensive multigene panel investigation of hearing loss in Brazil to date. By bridging the gap between clinical phenotyping and advanced genomics, we have demonstrated that: (i) Broad panels are superior for identifying “non‐syndromic mimics.” (ii) Population‐specific data is essential for equitable precision medicine. (iii) Early molecular diagnosis transforms clinical management from reactive to predictive.

Our findings advocate for expanding access to genomic testing in populations that remain underrepresented in global genetic reference databases, ensuring that the benefits of the genomic revolution are more equitably distributed.

## Funding

This work was supported by Fundação de Amparo à Pesquisa do Estado de São Paulo, 2023/07188‐7. BGI GENOMICS.

## Conflicts of Interest

The authors declare no conflicts of interest.

## Supporting information


**Table S1:** Comprehensive data on candidate, resolved, and inconclusive variants, alongside the clinical features of the subjects carrying them. Classification of all variants associated with resolved, candidate, and inclusive cases, including information regarding ClinGen curation of each gene and ACMG classification of the variants. Novel variants are underlined. Alleged IP refers to the presumed inheritance pattern before molecular diagnosis. DVD: Deafness Variation Database, NSHL: Non‐syndromic hearing loss, P: Pathogenic, LP: Likely Pathogenic, VUS: Variant of Unknown Significance, N/A: not available, Zyg.: Zygosity, HET: heterozygous, HOM: homozygous, Pathogen.: ACMG Classification regarding pathogenicity; * cases with more than one candidate variant, # cases also monoallelic to *GJB2* recessive variants.

**Table S2:** Summary of Genetically Resolved Cases (*n* = 43). Recurrent genes are indicated in bold.

## Data Availability

The datasets generated and analyzed during the current study are not publicly available due to privacy and ethical restrictions on human genomic data, as approved by the HC‐FMUSP Ethics Committee. However, relevant variant data have been deposited in ClinVar [accession numbers provided upon publication]. De‐identified sequencing data and the custom bioinformatics pipeline are available from the corresponding author on reasonable request for scientific peer review or collaboration. Regional allele frequencies utilized in this study are publicly accessible via the AbraOM database (http://abraom.ib.usp.br/).
