# Supplementary material for: Genetic Landscape of Hearing Loss in Brazilian Patients Reveals Population‐Specific Variants and Clinical Correlations
Source: Clin Genet. 2026 Jun 3;110(2):210–26. doi: 10.1111/cge.70186 (PMC13327172; doi:10.1111/cge.70186)
Supplement: Supplementary file 2 — Table S2: Summary of Genetically Resolved Cases (n = 43). Recurrent genes are indicated in bold. [file CGE-110-210-s002.docx]

Table S2: Summary of Genetically Resolved Cases (n=43). Recurrent genes are indicated in bold.

| **Inheritance** | **Phenotype** | **Genes Identified** | **Key Clinical Notes** |
| --- | --- | --- | --- |
| Autosomal Recessive (60.5%) | Non-Syndromic | ***MYO15A, CDH23, CLDN14, LRTOMT, TMC1, OTOGL***, *CLDN14, LRTOMT, EPS8, MPZL2, OTOF, RDX, TMPRSS3, USH1C* | Most common cause of prelingual SNHL in this cohort. |
|  | Syndromic | ***MYO7A****, USH2A, CLRN1, PCDH15, CDH23, PEX6, BSND* | Includes Usher Syndrome (Type 1 & 2) and Heimler Syndrome Type 2. |
| Autosomal Dominant (39.5%) | Non-Syndromic | ***GSDME, MYO7A, MYO6, P2RX2****, MYH9, PTPRQ, EYA4, ACTG1* | Higher frequency of postlingual onset and progressive loss. |
|  | Syndromic | MITF, PAX3, EYA1 | Includes Waardenburg Syndrome and Branchio-otic Syndrome Type 1. |
